# Modulation of Postprandial Plasma Concentrations of Digestive Hormones and Gut Microbiota by Foods Containing Oat ß-Glucans in Healthy Volunteers

**DOI:** 10.3390/foods12040700

**Published:** 2023-02-06

**Authors:** Martin Gotteland, Alejandra Zazueta, José Luis Pino, Andrea Fresard, Verónica Sambra, Juana Codoceo, María José Cires, Ximena López, Juan Pablo Vivanco, Fabien Magne

**Affiliations:** 1 Department of Nutrition, Faculty of Medicine, University of Chile, Independencia, Santiago 8380453, Chile; 2 Laboratory of Microbiology and Probiotics, Institute of Nutrition and Food Technology (INTA), University of Chile, Macul, Santiago 7830489, Chile; 3 Microbiology and Mycology Program, ICBM, Faculty of Medicine, University of Chile, Independencia, Santiago 8380453, Chile; 4 Consorcio de Cereales Funcionales (CCF), Huechuraba, Santiago 8590871, Chile; 5 Granotec Chile S.A., Huechuraba, Santiago 8590871, Chile; 6 Department of Food Science and Chemical Technology, Faculty of Chemical and Pharmaceutical Sciences, University of Chile, Independencia, Santiago 8380494, Chile

**Keywords:** beta-glucan, gut microbiota, digestive hormones, appetite, post-prandial glycemia

## Abstract

Cereal β-glucans are beneficial health ingredients that reduce cholesterolemia and postprandial glycaemia. However, their impact on digestive hormones and gut microbiota is not yet fully established. Two randomized, double-blind, controlled studies were conducted. In the first study, 14 subjects ingested a breakfast with or without β-glucan from oats (5.2 g). Compared to the control, β-glucan increased orocecal transit time (*p* = 0.028) and decreased mean appetite score (*p* = 0.014) and postprandial plasma ghrelin (*p* = 0.030), C-peptide (*p* = 0.001), insulin (*p* = 0.06), and glucose (*p* = 0.0006). β-glucan increased plasma GIP (*p* = 0.035) and PP (*p* = 0.018) without affecting leptin, GLP-1, PYY, glucagon, amylin, or 7α-hydroxy-4-cholesten-3-one, a biomarker of bile acid synthesis. In the second study, 32 subjects were distributed into 2 groups to ingest daily foods with (3 g/day) or without β-glucan for 3 weeks; stools were collected before/after treatment. No changes in fecal microbiota composition/diversity (deep sequencing) were detected with β-glucans. These results indicate that acute intake of 5 g β-glucan slows transit time and decreases hunger sensation and postprandial glycaemia without affecting bile-acid synthesis, these changes being associated with decreased plasma insulin, C-peptide, and ghrelin, and increased plasma GIP and PP. However, regular daily intake of 3 g β-glucan is not sufficient to have an effect on fecal microbiota composition.

## 1. Introduction

Cereal β-glucans are soluble and linear polymers of glucose that are abundant in the cell wall of oats and barley. Their high molecular weight (1000–2500 kDa) and solubility determine their viscosity in solutions and their physiological effects in humans [1,2]. Due to their physicochemical characteristics, β-glucans have been used as fat substitutes to reduce the caloric content of foods [3,4,5]. They also form viscous solutions in the gastrointestinal lumen, contributing to slowing gastric emptying and increasing feelings of fullness and satiety [6]. In addition, they also interfere with enzymatic activities and bile micelles [7], decreasing postprandial glucose absorption and increasing fecal excretion of bile salts, forcing the body to synthesize new bile acids at the expense of endogenous cholesterol [2,8]. Accordingly, several clinical studies have reported that the consumption of foods containing β-glucans contributes to the maintenance of normal blood cholesterol and to the reduction of postprandial hyperglycemia in human subjects [8,9]. Based on this background, the European Food Safety Authority (EFSA) has accepted health claims for β-glucans from cereals. These claims state that the intake of at least 3 g and 4 g of cereal β-glucan may improve blood cholesterol and postprandial glycaemia, respectively [10].

The administration of oat β-glucans was also shown to stimulate the release of cholecystokinin (a digestive, anorexigenic hormone) in overweight women, in association with decreased insulin release and increased subjective satiety [11]. However, their impact on the level of other digestive hormones involved in the regulation of appetite, gastric emptying and/or metabolism, such as GLP-1, GIP, PYY, PP, leptin, and ghrelin, has been less well studied. Although plasma ghrelin and PYY decreased and increased, respectively, after a meal enriched with oat bran, no changes in the release of digestive hormones, appetite, or energy intake were observed in healthy young subjects after ingesting different doses of dietary fiber in another study [12,13]. On the other hand, Weickert et al. reported that wheat intake, but not that of β-glucan from oats, affects the postprandial secretion of PYY and ghrelin [14]. The results regarding the effect of β-glucans on satiety-regulating hormones are therefore contradictory, and further studies are needed to reach a consensus. Finally, recent in vitro and in vivo studies suggest that β-glucans may also act as prebiotics, modulating the colonic microbiota and stimulating the production of short-chain fatty acids (SCFAs) through their fermentation [15]. However, most studies evaluated the effect of β-glucans on the growth of *Lactobacillus* or *Bifidobacterium* species in pure cultures, the composition of human fecal microbiota in bioreactors, or the cecal microbiota in rats. Few studies were performed in humans and most did not analyze the whole microbiota, but only some specific bacterial populations through plate count, qPCR, or fluorescent in situ hybridization (FISH) [15].

Based on these antecedents, the aim of this study was to determine, in asymptomatic human volunteers, (1) the acute effect of a breakfast enriched with oat β-glucan on orocecal transit time, changes in plasma digestive hormones, and satiety; and (2) the effect of a three-week intake of foods enriched with oat β-glucan on the composition and diversity of gut microbiota, assessed by deep sequencing.

## 2. Materials and Methods

### 2.1. Subjects

The study was conducted at the Department of Nutrition of the Faculty of Medicine, University of Chile. The protocol was approved by the “Comité de Etica de Investigacion en Seres Humanos” (CEISH) (Acta 145-2014) of the Faculty of Medicine, the subjects were informed about the objectives and procedures of the study, and those who agreed to participate had to sign a written consent. Asymptomatic subjects, between 20 and 40 years of age, male or female, normal weight or overweight (BMI between 18.5 and 29.9 kg/m^2^) were recruited. Exclusion criteria included pregnancy, history of digestive diseases, cholecystectomy, chronic intestinal pathologies and/or malabsorption syndrome (celiac disease, chronic inflammatory bowel diseases), as well as the intake of drugs that interfere with the intestinal microbiota or intestinal transit (antibiotics, anti-inflammatory drugs, laxatives, and prokinetics) during the month prior to the study. Smoking, type-2 diabetes, organ failure (cardiac, hepatic, renal, and respiratory), or immunodeficiency (HIV, chemotherapy, radiotherapy, and transplantation) were further exclusion criteria. A biochemical and lipid profile was performed on the subjects recruited to eliminate those who might present alterations incompatible with the study.

### 2.2. Food Products

Different foods (soup, lactose-free yoghurt, lactose-free milkshake, orange nectar, cereal bars, and biscuits) were developed for use in short and long-term studies. Those used by the β-glucan group were supplemented with an enriched beta-glucan fraction obtained from dehulled oat groats by fine grinding and air fractionation (BETAvena, Granotec, Chile), a technological process that allows oat beta-glucans to remain in their native form. The molecular weight of β-glucans ranged from 65 to >2000 kDa. The viscosity of this beta-glucan concentrate in solution was not determined in this study. All β-glucan-enriched foods contained 1 g of β-glucans per serving. Control subjects were given the same foods without beta-glucans.

### 2.3. Short-Term Study

#### 2.3.1. Experimental Design

The change in the area under the curve (AUC) of glycemia was chosen as the primary outcome to calculate the sample size. To detect a 20% decrease in this parameter with β-glucan in the setting of a crossover study, with a power of 80% and a risk α of 5%, 14 subjects had to be recruited. An experimental, randomized, double-blind, controlled, crossover trial comprising two days of testing (β-glucan and control) separated by at least one week was performed. Each subject received advice from a dietician to avoid as much as possible foods containing oat or barley and changes in their diet between the two test periods. On each of the test days, subjects who fasted overnight were required to report to the Nutrition Department at 8:00 am. An intravenous catheter was placed in the forearm vein and two basal blood samples were taken 10 min apart. Subjects were then required to eat a breakfast consisting of 200 mL of lactose-free milkshake, 2 cookies, and 2 cereal bars, with or without β-glucans (β-glucan and control period, respectively), in a time not exceeding 15 min. The nutritional composition of the breakfasts is described in Table 1. The breakfast enriched with β-glucan provided 5.2 g of β-glucan. This amount of β-glucan was chosen on the basis that the intake of at least 4 g of cereal β-glucan can improve postprandial blood glucose levels and that most of the outcomes assessed in this short-term study were related to glucose metabolism.

Blood samples were obtained 30 min, 1:30, 3:30, 5:30, and 7:30, after finishing breakfast. At the end of the study day, volunteers were offered a sandwich and a fruit juice. The food products were prepared and provided by the “Consorcio de Cereales Funcionales” in Santiago and delivered to the laboratory at the beginning of the study. The control and experimental products were individually packaged and had an identical appearance, each being labeled with a code that allowed them to be differentiated, but whose identity was unknown to both the researchers and the volunteers. 

#### 2.3.2. Orocecal Transit Time Determination

A Hydrogen Breath test (HBT) was performed on the volunteers during the test, to determine their orocecal transit time (OCTT). Breath samples were obtained by end expiratory sampling in plastic syringes using a modified Haldane-Priestley tube, before breakfast ingestion and at 15-min intervals thereafter. The hydrogen concentration in breath samples was measured using an electrochemical cell (Lactotest, Medical Electronic Construction, Brussels, Belgium). OCTT was defined as the time elapsed between the start of breakfast and that at which an increase of more than 20 ppm above baseline H_2_ occurred [16].

#### 2.3.3. Satiety Index

Sensations of hunger (how hungry are you?), fullness (how full are you?), satiety (how satiated are you?), food craving (how strong is your desire to eat?), and prospective food consumption (how much would you be able to eat right now?) were assessed every 60 min for 8 h after breakfast intake in each subject using 10 cm visual analogue scales (VAS) [17]. Corresponding areas under the VAS curves (AUC, cm·min) were calculated to describe global changes in sensations during the post-prandial period. Average appetite (=desire to eat + hunger + (10-fullness) + prospective food consumption) was calculated according to Anderson et al. [18] to provide an overview of satiety.

#### 2.3.4. Plasma Hormones Determination

Blood samples were collected in EDTA tubes and a DPPIV inhibitor (Millipore) and protease inhibitor cocktail (Sigma) was immediately added, according to the manufacturer’s instructions. Samples were centrifuged at 1000× *g* for 10 min and the plasma was aliquoted and stored at −30 °C. A Human Metabolic Hormone Magnetic Bead Panel (HMHEMAG-34K, Milliplex, Merck, Santiago, Chile) [19] was used to simultaneously determine plasma concentrations of ghrelin, leptin, gastric inhibitory polypeptide (GIP), glucagon-like peptide 1 (GLP-1), peptide YY (PYY), pancreatic polypeptide (PP), glucagon, amylin, insulin, and C-peptide, using a Luminex 200 System (Merck, Santiago, Chile), according to the manufacturers’ instructions. 

Post-prandial glucose concentrations were measured at 0, 30, 60, and 120 min with a glucometer (Accu-Check, Roche, Santiago, Chile) and plasma 7α-hydroxy-4-cholesten-3-one (7α-HC) was determined by liquid chromatography coupled mass spectrometry (LC-MS/MS) by the Clinical Laboratory of the Pontificia Universidad Católica (Santiago, Chile) (intra-assay coefficient of variation = 5.6%) [20].

### 2.4. Long-Term Study

#### 2.4.1. Experimental Design

The primary outcome selected for the sample size calculation was the relative abundance of butyrate-producing bacteria in the fecal microbiota of the subjects. Considering that these bacterial populations represent about 10% of the total microbiota, to have 90% chance of detecting a 5% increase in these populations with a 5% risk and considering a 10% dropout, it was estimated that it was necessary to recruit 16 subjects per group. An experimental, randomized, double-blind, controlled clinical study was conducted. The recruited subjects were randomly distributed into two groups: control and β-glucan. Different foods (soup, lactose-free yogurt, lactose-free milkshake, orange nectar, cereal bars, and cookies) enriched with β-glucan (β-glucan group) or without β-glucan (control group) were provided to the subjects weekly for three weeks, under the supervision of a registered dietitian. All β-glucan-enriched foods contained 1 g of β-glucan per serving and subjects had to ingest 3 servings per day. The volunteers were free to eat other foods during the day but were asked not to consume oats or barley-containing products. Each volunteer had to deliver a freshly emitted stool in a plastic container before the beginning (baseline T0) and at the end (T1) of the treatment period. The stools were kept frozen until analysis.

#### 2.4.2. Digestive Symptoms

During the study, volunteers had to register daily the eventual presence of digestive symptoms (abdominal pain, abdominal distension, vomiting/regurgitation, increased borborygmi, and increased rectal gas) and distractors, and their respective intensity (0: absent, 1: low, 2: mild, and 3: high), as previously described [21]. They also had to register their stool frequency and consistency daily according to the seven-point Bristol stool scale, using an ad hoc form. For the statistical analysis, the sum of the digestive symptoms was calculated for each study week and for each subject considering their respective intensity.

#### 2.4.3. Microbiota Analysis

Bacterial genomic DNA was extracted from 220 mg of stool samples using the QIAmp DNA Stool Mini Kit (Qiagen, Hilden, Germany) according to manufacturer instructions. Library preparation and Illumina sequencing were performed at the Roy J. Carver Biotechnology Center, University of Illinois (Urbana-Champaign, Champaign, IL, USA). Libraries were prepared from 2 ng of DNA using the Fluidigm Access Array (Fluidigm, South San Francisco, CA, USA) in a two-step process. In the first step, the V3–V4 region of the 16S rRNA gene was amplified using the primers 341F (50-CCTACGGGNGGCWGCAG-30) and 785R (50-GACTACHVGGGTATCTAATCC-30) [22], and index and sequencing adapters were added in a second PCR. The amplicons were quantified through Qubit fluorometry, and their sizes were verified in 11 random samples using an Agilent 2100 Bioanalyser (Agilent Technologies, Santa Clara, CA, USA) to determine their overall quality. The amplicons were then pooled, purified with a 2% agarose e-gel (Invitrogen, Life Technologies, Grand Island, NY, USA), and the average amplicon size was determined. Finally, pooled libraries were quantified with qPCR performed using a CFX connect Real-Time PCR (Bio-Rad, Hercules, CA, USA) before loading the libraries into the sequencer. Sequencing was performed with MiSeq Illumina system (Illumina, San Diego, CA, USA), using the V3 kit, generating paired end reads of 2300 nt.

Illumina FASTQ sequences were analyzed with the QIIME software package, as previously described [23]. Paired reads were demultiplexed (CASAVA V1.8.2), trimmed (Trimmomatic V0.36) to remove low-quality sequences, merged (FLASH V1.2.11), and adapters removed (Cutadapt V1.9). Chimeric sequences were removed with VSEARCH. OTUs were constructed as described in the closed reference protocol in QIIME (V 1.8.0) using the the Greengenes 13.8 database at 97% sequence similarity. Rarefactions curves were calculated using QIIME. All analyses of abundances and α- and β-diversity were performed using the Phyloseq and microbiome packages in R statistical software. Values were obtained for observed OTUs and Chao1 index.

### 2.5. Statistical Analysis

Except for the results corresponding to the microbiota analysis, which were processed using R statistical software [24], all other data were analyzed using “Statistica” (StatSoft, Tulsa, OK, USA). Whether the variables followed a normal distribution was assessed with the Shapiro-Wilks test. Results were expressed as mean ± SD or as median [interquartile range]. Changes in the variables between the initial and final periods of both groups were analyzed by analysis of variance.

## 3. Results

### 3.1. Short-Term Study

Of the 18 asymptomatic volunteers initially recruited in the acute study, 4 were excluded because they presented alterations in their biochemical profiles (fasting glycemia >110 mg/dL) incompatible with their participation. The remaining 14 subjects completed the study; their anthropometric characteristics and biochemical/lipid profiles are described in Table S1.

**Figure 1:**
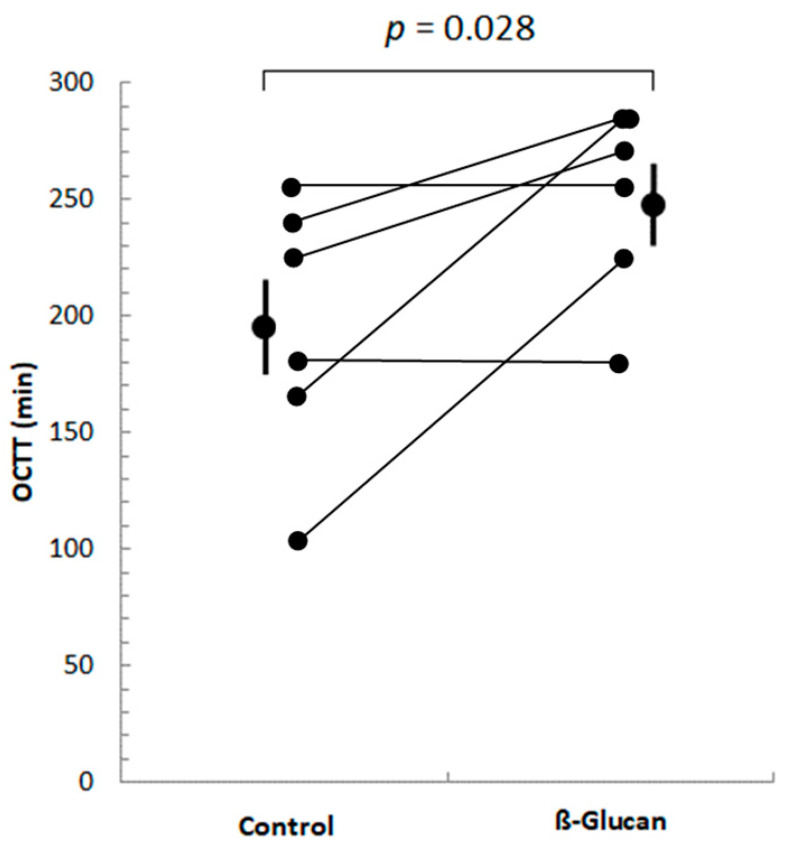
Orocecal transit time (OCTT) of volunteers after ingestion of control or β-glucan breakfast. Only six subjects showed post-prandial increases in their breath H_2_ > 20 ppm above baseline H_2_ values in both periods. The mean OCTT values (±SEM) of these 6 subjects are shown. OCTT increased significantly (*p* = 0.028) in the ß-glucan period compared to the control period.

Of these 14 participants, only 6 were identified as hydrogen producers, i.e., they showed increases in breath H_2_ concentrations greater than 20 ppm above baseline values in both breath test periods, compared with baseline values, allowing their OCTT to be determined. Five subjects had an increase in H_2_ in the control period and not in the β-glucan period, and only one had an increase in H_2_ in the β-glucan period and not in the control period. As shown in Figure 1, OCTT increased significantly (by 28%) in all 6 subjects during the β-glucan period, compared to the control period. 

**Figure 2:**
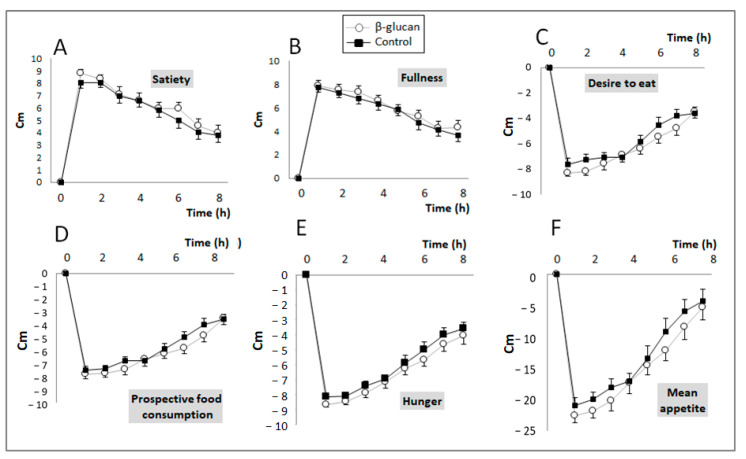
Changes in subjective feelings (VAS score) of satiety/appetite during the postprandial period after eating breakfast with foods enriched (Grey line) or not (black line) with β-glucan. (**A**) Satiety (Treatment (Trt.) × Time: *p* = 0.65); (**B**) Fullness (Trt. × Time: *p* = 0.95); (**C**) Desire to eat (Trt. × Time: *p* = 0.29); (**D**) Prospective food consumption (Trt. × Time: *p* = 0.39); (**E**) Hunger (Trt. × Time: *p* = 0.97); (**F**) Mean appetite score (Trt. × Time: *p* = 0.83). Means ± SEM. Two-way ANOVA for repeated measurements (Treatment × time interaction).

Post-prandial feelings of hunger, fullness, satiety, food craving, and prospective food consumption were evaluated by VAS scoring during the control and β-glucan periods, and the mean appetite score was calculated according to the values obtained. The VAS results are shown in Figure 2; no difference was observed between the two groups for any of the parameters studied. 

However, when the results were expressed as AUCs for each of these variables (Table 2), paired comparisons indicate that breakfast with β-glucan-enriched foods non-significantly decreased “desire to eat” (*p* = 0.05) and significantly decreased hunger and prospective food consumption (*p* = 0.04 and *p* = 0.033, respectively). Accordingly, the subjects’ mean appetite score also significantly decreased in the β-glucan period (*p* = 0.014).

**Figure 3:**
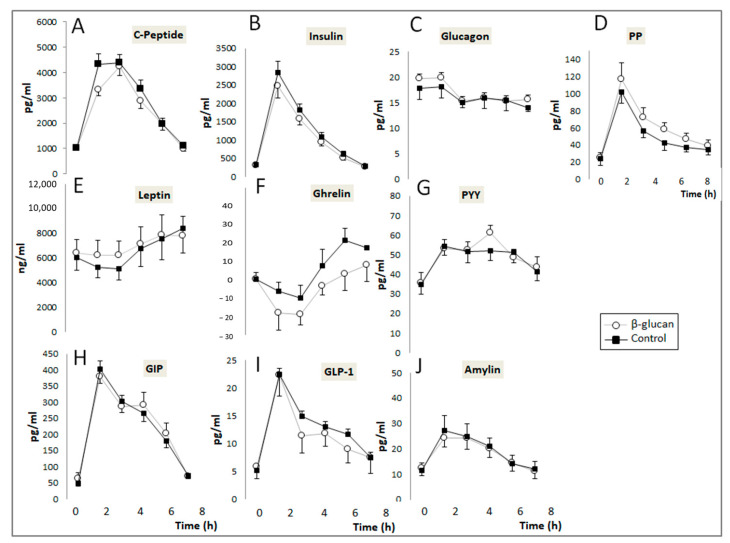
Post-prandial changes in the plasma concentrations of digestive hormones. (**A**–**C**) peptide (Treatment (Trt.) × Time: *p* = 0.75); (**B**) Insulin (Trt. × Time: *p* = 0.25); (**C**) Glucagon (Trt. × Time: *p* = 0.94); (**D**) PP (Trt. × Time: *p* = 0.83); (**E**) Leptin (Treatment (Trt.) × Time: *p* = 0.95); (**F**) Ghrelin (Trt × Time: *p* = 0.88); (**G**) PYY (Trt. × Time: *p* = 0.042); (**H**) GIP (Trt. × Time: *p* = 0.87); (**I**) GLP-1 (Trt. × Time: *p* = 0.79); (**J**) Amylin (Trt. × Time: *p* = 0.90). (Means ± SEM). Two-way ANOVA for repeated measurements (Treatment × time interaction).

Changes in the post-prandial plasma concentrations of digestive hormones and glucose are described in Figure 3. No significant Treatment X Time effect was detected when the postprandial control and β-glucan curves were compared for all digestive hormones evaluated in the study. However, paired comparisons of their corresponding AUCs (Table 3) indicate that administration of β-glucan non-significantly decreased the plasma concentrations of insulin by 18.8% (*p* = 0.06) and significantly decreased those of ghrelin by 1.5 time (*p* = 0.030) and C-peptide by 7.9% (*p* = 0.001). Intake of the β-glucan-enriched breakfast also increased plasma GIP and PP by 10.4% (*p* = 0.035) and 19% (*p* = 0.018), respectively, without affecting those of leptin, GLP-1, PYY, glucagon, and amylin. When considering differences in AUC between the β-glucan and control periods, a positive correlation was observed between insulin and C-peptide (r = 0.58; *p* = 0.03) and between PYY and GLP-1 (r = 0.75, *p* = 0.002). The AUC of blood glucose also decreased significantly by 9.5% (*p* = 0.006) after the β-glucan-enriched breakfast.

Plasma concentrations of 7-α-HC, used as a marker of cholesterol metabolism, did not differ at baseline between the two treatment periods (12.3 ng/mL [8.2–24.3] vs. 11.3 ng/mL [7.4–26.0], respectively, for the control and β-glucan period; *p* = 0.55). No differences in this parameter were observed 7 and 8 h after β-glucan ingestion, compared to the control period (ANOVA, *p* = 0.10).

### 3.2. Long-Term Study

Thirty-two subjects were enrolled in the study and their anthropometric characteristics as well as their biochemical and lipid profiles at inclusion are shown in Table S2. Both groups were similar in terms of the different parameters assessed, except for blood glucose and phosphatemia which were significantly higher and lower, respectively, in the β-glucan group than in the control group. All volunteers completed the study, and no adverse effects were reported.

The volunteers recorded the presence and intensity of digestive symptoms daily, including abdominal pain, bloating, borborygmi, rectal gas, and reflux/vomiting. The sum of these symptoms (total digestive symptomatology) by week for each group is shown in Figure S1. ANOVA shows a significant effect of treatment (*p* = 0.048), with digestive symptomatology being higher in the β-glucan group than in the control group. However, no time effect (*p* = 0.34) nor time X treatment interaction (*p* = 0.57) was detected. When individual digestive symptoms were compared between the two groups, no differences were detected for abdominal pain, borborygmi, rectal gas, and vomiting/regurgitation. Significantly more bloating was recorded in the β-Glucan than in the control group at week 1 (4 [1–7] vs. 0 [0–3], respectively; *p* = 0.04), but this difference disappeared at weeks 2 and 3. The weekly stool frequency, according to their consistency, for each treatment group is shown in Table S3. No changes in hard (corresponding to type 1 and 2 of the Bristol Scale) or watery (type 6 and 7) stool output were observed in the β-glucan group, compared to the control. Most of the stools emitted by the volunteers were of normal consistency (3 to 5), and their frequency varied between 5 and 7 per week, with no change over time or with treatment.

The fecal microbiota of the volunteers was characterized before and after the three-week period of dietary supplementation with β-glucan or control, by sequencing the V3–V4 region of the 16S RNA gene. Of the 32 subjects enrolled in the study, one from the control group and one from the ß-Glucan group were removed before analysis due to the low number of sequences (<8000) detected in their samples. A total of 1,548,315 high-quality filtered sequences were obtained, i.e., 25,805 ± 9033 sequences per sample. As shown in Figure S2, all four rarefaction curves reached an asymptote, indicating that the depth of the sequence was sufficient to represent most of the diversity of the bacterial community, no statistical differences were observed between them. 

**Figure 4:**
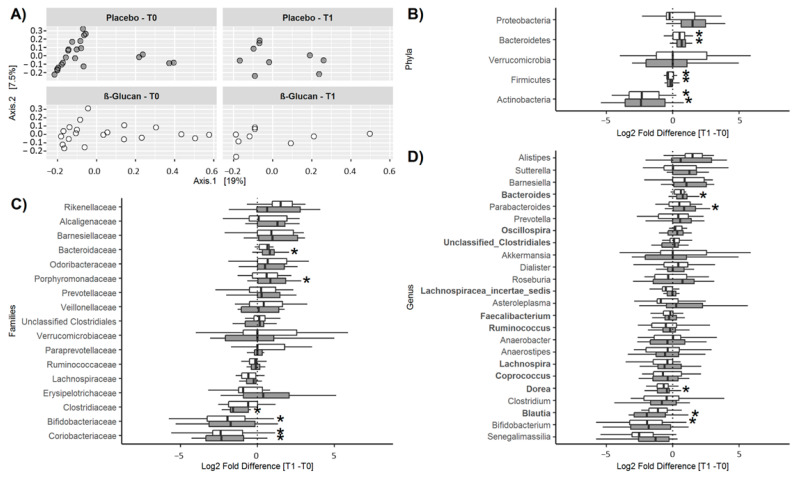
ß-diversity and changes in relative abundances (Log2 fold differences) of bacterial taxa between T1 and T0. (**A**) Inter-individual β-diversity at T0 (before treatment) and T1 (after treatment) in the control and β-glucan groups. Changes in the relative abundances of bacterial phyla (**B**), families (**C**), and genera (**D**). In (**B**–**D**), only bacterial taxa with a prevalence >50% are shown. In the box and whisker plots, the line shows the median, the wide of the box, the interquartile range and the whiskers, the highest and lowest values. * *p* < 0.05.

A total of 10 phyla, 44 families, and 79 genera were detected. The core microbiota representing the genera present in all subjects at baseline included *Faecalibacterium* (25.9 ± 6.1%), *Lachnospiracea incertae sedis* (10.9 ± 4.0%), *Blautia* (4.9 ± 2.8%), unclassified clostridiales (4.2 ± 2.8%), *Coprococcus* (3.2 ± 1.3%), *Ruminococcus* (1.7 ± 1.2%), *Lachnospira* (1.2 ± 1.1%), *Dorea* (1.0 ± 0.6%), and *Oscillospira* (0.89 ± 0.61%), all belonging to the phylum Firmicutes, and *Bacteroides* (17.8 ± 11.4%) of the phylum Bacteroidetes. As shown in Figure 4A, no significant differences in diversity were detected between the two groups at T0 or T1. Furthermore, α-diversity was similar in both groups at T0 (Observed: 846 ± 147 vs. 836 ± 108; Chao-1: 1456 ± 271 vs. 1457 ± 246, for the control and β-glucan group, respectively) and did not change significantly at T1 (ANOVA: *p* = 0.29 and *p* = 0.26 for Observed and Chao-1). No differences in α-diversity were observed between genders (*p* = 0.33 and *p* = 0.64 for the Observed and Chao-1 index, respectively). Relative abundances of the phyla, families, and genera at T0 and T1 are shown in Table S4. Only taxa with a relative abundance >0.1% were considered. The corresponding changes between T1 and T0 are shown in Figure 4B–D. Significant changes were observed at T1, compared to T0, for some bacterial populations. At the phylum level (Figure 4B), the abundances of Actinobacteria and Firmicutes decreased and that of Bacteroidetes increased over time in both groups, with no differences between them. Considering changes over time at the family level (Figure 4C), the abundance of Bacteroidaceae and Porphyromonadaceae increased and that of Clostridiaceae decreased only in the control group, while that of Bifidobacteriaceae decreased in the β-glucan group and that of Coriobacteriaceae decreased in both groups, these changes not being significantly different between groups. Finally, considering changes at the genus level (Figure 4D), the abundance of *Bacteroides* and *Parabacteroides* increased and that of *Dorea* and *Blautia* decreased, only in the control group, while that of *Bifidobacterium* decreased in the β-glucan group. Again, these changes over time were not significantly different between the groups. In summary, no significant differences were observed between the control and the β-glucan group for these different taxa at the end of the treatment period.

## 4. Discussion

The worldwide increase in obesity, type-2 diabetes, non-alcoholic liver disease, and cardiovascular diseases has stimulated interest in identifying dietary constituents capable of controlling blood glucose, insulin, and lipids as well as blood pressure, and food intake. Dietary fibers, including β-glucans, have been implicated in the prevention of insulin resistance, hypertension, dyslipidemia, and obesity, although the exact mechanisms associated with these health benefits have not yet been fully elucidated.

In the present study, we evaluated the effect of β-glucan intake on plasma concentrations of 10 digestive hormones simultaneously in healthy subjects, in addition to the determination of hunger perception, orocecal transit time, post-prandial glycaemia and plasma 7-α-HC, a marker of cholesterol metabolism. Our results show that acute ingestion of a breakfast containing 5.2 g β-glucan from oats leads to a significant decrease in the mean appetite score of volunteers during the postprandial period, mainly due to reduced hunger sensation and prospective food intake. Similar to our results, most studies conducted with β-glucans in human volunteers showed a positive effect on hunger/satiety sensation, although their real impact on subsequent food and energy intake was less evident [25,26,27,28]. Only a few studies did not show a positive effect of β-glucan on hunger perception. In the case of the Peters et al. study [29], for example, it is probable that this lack of effect was due to the low amount of β-glucan (1.2 g) administered to the volunteers. 

Our results also confirm that breakfast with β-glucans decreased post-prandial glycaemia and insulinemia, as reported in other studies [1,6,9,10,30]. Such effect could be explained by the fact that β-glucans slow gastric emptying [6], interfere with amylase activity [31], and reduce the expression of the glucose transporters SGLT-1 and GLUT2 at the brush border of enterocytes [32], through their ability to form viscous solutions in the lumen of the upper GI tract [12]. In our study, the impact of β-glucan breakfast on gastric emptying, and eventually on intestinal transit time, is suggested by the slower OCTT observed in the hydrogen-producing subjects and could also contribute to the reduced sensation of hunger/satiety. Regarding the insulin response elicited by breakfast with β-glucan, we only detected a non-significant decrease in insulin release, as reflected by changes in plasma insulin and AUC. However, the AUC of C-peptide was clearly decreased (*p* = 0.001). Since the pancreas releases C-peptide from pro-insulin in equimolar amounts and its hepatic extraction rate is lower than that of insulin, it is considered a reliable marker of insulin secretion [33]. This is confirmed by the fact that changes (between the β-glucan and control periods) in plasma insulin correlated with those observed with C-peptide. Consequently, our results indicate a decrease in insulin secretion, although plasma insulin levels were not significantly affected (*p* = 0.06). This lower pancreatic insulin response with β-glucan breakfast is probably due to the slower glucose absorption observed in our study, which would result in less stimulation of insulin release by the pancreas. These results highlight the importance of simultaneously determining insulin and C-peptide. 

Regarding post-prandial changes in the other digestive hormones, we observed a significant decrease in post-prandial ghrelin and an increase of GIP and PP with β-glucan breakfast, while leptin, GLP-1, PYY, glucagon, and amylin were not affected. GLP-1, PYY, leptin, and PP are anorexigenic hormones while ghrelin is orexigenic. In addition, amylin, which is released by pancreatic β-cells, may also act in the brain, producing satiety-like effects [34]. Consequently, all these hormones are involved in the highly complex process of appetite/satiety regulation [35]. Regarding our results, it is likely that the decrease in plasma ghrelin and the increase in PP help to explain the lower mean appetite score observed after breakfast with β-glucan, in the absence of changes in the other anorexigenic hormones. Although several studies have assessed the impact of β-glucan on digestive hormones, they did not determine as many hormones simultaneously as in our study. Our results confirm those reported by Barone Lumaga et al. who also observed a reduction in plasma ghrelin and an increase in PP with the intake of a drink containing 3 g of β-glucan in healthy volunteers, with no changes in PYY, GLP1, and GIP [36]. Vitaglione et al. [37] and Juvonen et al. [12] also reported reduced ghrelin levels with bread or pudding containing β-glucan, respectively, while in both studies, an increase in PYY levels was also observed, in opposition to our observations. In another study, ingestion of a whole-meal rye bread containing oat β-glucan concentrate resulted in a decrease of GIP with no change in GLP-1 [38]. Some studies were also conducted in patients with obesity or metabolic syndrome, showing that acute administration of β-glucan enriched foods dose-dependently increases PYY [39], increases CCK without affecting ghrelin (8), or decreases GIP without affecting insulin, GLP-1, or ghrelin [40]. Beck et al. also demonstrated that β-glucan supplementation for three months in overweight women on an energy-deficient diet resulted in decreased plasma leptin, PYY, and GLP-1 levels and increased CCK [41]. In contrast to other studies, we observed increased plasma levels of GIP after breakfast with β-glucan. GlP is mainly secreted by enteroendocrine K-cells located in the proximal gut epithelium, which explains the rapid onset of its secretion after a meal. Dietary fat has been shown to be the most potent stimulator of GIP secretion in humans [42]. Consequently, it is possible that the higher levels of GIP observed in our study are due to the fact that the lipid content of the β-glucan breakfast was slightly higher than that of the control, a difference inherent to the elaboration of the β-glucan-containing foods to maintain good acceptability of these products. In addition to its well-described role as an incretin hormone acting on pancreatic β-cells and in the control of lipid metabolism in adipose tissue, GIP has recently been shown to regulate progenitor cell proliferation in the central nervous system, behavior, and bone remodeling [42]. 

Moreover, we also determined post-prandial changes in plasma 7α-HC, a metabolic intermediate in bile acid synthesis. Bile acid synthesis in the liver and its fecal excretion are key events in the regulation of endogenous cholesterol pool. Bile acid excretion has been reported to increase significantly within 24 h of ingestion of oat β-glucans, a phenomenon that results in the stimulation of bile acid synthesis with the formation of 7α-HC. Serum 7α-HC correlates with the activity of cholesterol 7-α-hydroxylase, and the rate-limiting liver enzyme for bile acid synthesis and is therefore considered a reliable marker of bile acid synthesis in humans [43]. Andersson et al. [44] reported an increase in plasma 7α-HC by 84% 8 h after ingestion of a breakfast containing 11 g of oat β-glucan, i.e., a very high amount of this dietary compound. In our study, no changes in plasma 7α-HC were detected at 7 and 8 h postprandially, probably because the breakfast given to our volunteers provided only 5.2 g of β-glucan. These results suggest, therefore, that this dose of β-glucan, when consumed acutely, is insufficient to stimulate bile acid synthesis. 

We also conducted a second clinical study to assess the prebiotic effect of daily consumption of 3 g of β-glucan from oat for 3 weeks in human volunteers. We selected this amount because it is the daily intake suggested by EFSA for functional foods containing β-glucan to reduce cholesterolemia in humans, and we wanted to see if this lower amount could change the microbiota. The composition and diversity of the fecal microbiota were determined by high-throughput sequencing before and at the end of the treatment period in both groups. Our results suggest that daily intake of 3 g of β-glucan for 3 weeks does not significantly affect fecal microbiota diversity and composition. Although several in vitro, animal and human studies have evaluated the impact of β-glucan on bacterial growth and microbiota composition, their results are highly contradictory, and it is still difficult to draw firm conclusions about the possible prebiotic effect of β-glucans.

For example, β-glucan from barley was shown to stimulate the growth of *B. infantis, B. longum,* and *B. adolescentis* in pure culture in a 24 h batch fermentation [45]. These results were confirmed by Shen et al. [46] who reported a dose-dependent increase in *Lactobacillus* and *Bifidobacterium* in association with a decrease of Enterobacteriaceae in rats supplemented with cereal β-glucan for 6 weeks. However, another study using human fecal microbiota cultured in a fermenter with β-glucan showed higher levels of the family Erysipelotrichaceae and the genus *Syntrophococcus,* and lower levels of clostridiales, with no changes in bifidobacteria [47]. Similarly, Hughes et al. reported no significant changes of *Lactobacillus* and *Bifidobacterium* populations with β-glucan inoculated under similar experimental conditions. These authors described that β-glucan fermentation led to an increase in the *C. histolyticum* cluster and, to a lesser extent, up-regulation of clostridia cluster IX, *Bacteroides–Prevotella*, and *Atopobium*, these changes being accompanied by the generation of SCFAs characterized by a high propionate content [48]. 

Few clinical trials have evaluated the impact of β-glucans on the composition of the microbiota. In a study of 20 polypectomized patients consuming 125 g/day of bread with 3 g ß-glucans for 3 months, Turunen et al. analyzed fecal total aerobes and anaerobes, coliforms, *E. coli*, *Enterococcus*, *C. perfringens*, *Bacteroides* species, *Bifidobacterium* species, *Lactobacillus* species, and *Candida* species by plate count at baseline, and one and three months. Only slight changes were observed: significant decreases in total coliform at day 30 and *C. perfringens* at day 90, with no changes in *Bifidobacterium* and *Lactobacillus* [49]. Consequently, fecal SCFA content was hardly changed in these subjects and no changes in fecal β-glucuronidase and β-glucosidase activities and pH were detected. On the other hand, Mitsou et al. recruited 52 healthy volunteers who had to ingest a cake with 0.75 g of β-glucan daily. Using culture methods, they reported an increase of *Bifidobacterium* species after 15 d of treatment, but only in subjects over 50 years of age [50]. However, these results are questionable considering that the treated group had significantly lower *Bifidobacterium* counts than the control group at the start of the treatment. In another clinical trial, Nilsson et al. [51] determined changes in fecal SCFAs in healthy volunteers supplemented with 20 g of dietary fibers including 10 g β-glucan daily for 8 weeks. They reported an increase in acetic, propionic, butyric, isobutyric, and isovaleric acids, and a decrease in lactic acid, compared to baseline, suggesting that β-glucans were fermented in the colon. However, no control group was used in this study.

Finally, only two studies used high-throughput sequencing to study changes in the microbiota following β-glucan intake. In the first study, the fecal microbiota of 26 healthy subjects was compared before and after a 2-months dietary intervention with pasta containing 3 g of barley β-glucans [52]. No changes in diversity were observed after β-glucan intake, and pyrosequencing results indicated that the relative abundances of Eubacteriaceae, Ruminococcaceae, and Fusobacteriaceae families and *Clostridium* and *Faecalibacterium* genera decreased at the end of the treatment period. Using culture methods, the authors also reported higher counts of *Lactobacillus* species and lower counts of *Bacteroides*/*Prevotella*, Enterobacteriaceae, and total coliforms at the end of the treatment. No changes in *Bifidobacterium* were observed with both methods. However, a major drawback of this study was the absence of a control group, which severely limits the interpretation of these results.

Recently, Wang et al. [53] conducted a double-blind, controlled, crossover study in 30 subjects who ingested successively, and in random order, for 5 weeks a control diet or a diet supplemented with 3 or 5 g/day of low molecular weight (LMW) barley β-glucan, or 3 g/day of high molecular weight (HMW) barley β-glucan. No changes in α-diversity were observed with β-glucan, regardless of its MW. Compared to the control period, the relative abundance of Firmicutes decreased and that of Bacteroidetes increased with 3 g of HMW β-glucan, with no change in response to 5 g HMW or 3 g LMW β-glucan. At the lower taxonomical level, the order Bacteroidales and the genera *Bacteroides* and *Streptococcus* increased in the period with 3 g/day HMW β-glucan. A limitation of this interesting study is that the microbiota was analyzed only at the end, and not at the beginning of the four treatment periods. Although these were separated from each other by a 4-week washout period, it is unclear whether this time was sufficient to allow the microbiota to restore its initial composition. In summary of these studies, the impact of β-glucan administration of on the microbiota appears to be very modest, compared with other dietary fibers such as fructo- and galacto-oligosaccharides whose prebiotic properties have been well studied. 

## 5. Conclusions

This study confirms the interest in using β-glucans for functional foods, due to their beneficial effects on gastrointestinal transit time, post-prandial levels of blood glucose and digestive hormones, and satiety. However, further studies are needed to determine what levels of β-glucan intake are able to modulate gut microbiota in consumers. These results suggest that the health-promoting effects of dietary β-glucans (at the low dose of 3 g/d) are probably due more to their physiological effect in the proximal part of the gastrointestinal tract than to their prebiotic effect in the colon.

## Figures and Tables

**Table 1:** Nutritional composition of the breakfasts used in the study.

	Control	ß-Glucan
Energy (kcal)	393	396
Proteins (g)	9.5	11.6
Fat (g)	8.6	11.2
Available Carbohydrates (g)	69.5	62.1
Sugar (g)	12.7	11
Oat β-glucan (g)	0	5.2

**Table 2:** Area under the VAS curves (AUC) for satiety, fullness, hunger, food craving, prospective food consumption, and calculated values for mean appetite score during the control and β-glucan periods. Means ± SD.

AUC (cm·min)	Control (*n* = 15)	β-Glucan (*n* = 15)	*p* Value (Paired *t*-Test)
Satiety	47.1 ± 13.6	49.2 ± 12.9	0.19
Fullness	44.8 ± 12.6	46.8 ± 13.2	0.19
Desire to eat	−46.1 ± 12.4	−49.6 ± 11.0	0.05
Prospective food consumption	−45.0 ± 8.7	−48.3 ± 10.9	0.033
Hunger	−47.2 ± 9.0	−50.7 ± 9.1	0.04
Mean appetite score	−107 ± 39	−121 ± 39	0.014

**Table 3:** The area under the curves (AUC) of post-prandial plasma concentrations of digestive hormones and glucose during the control and β-Glucan periods (Means (CI_95%_)).

Post-Prandial AUC of Plasma Digestive Hormones (pg·h/mL)	Control (*n* = 14)	β-Glucan (*n* = 14)	*p* Value (Wilcoxon Paired Test)
Ghrelin	27 (−30–58)	−13.6 (−91–18)	0.030
Leptin	27,502 (13,502–38,172)	24,206 (16,115–47,404)	0.68
GIP	1204 (952–1513)	1329 (1161–1504)	0.035
GLP-1	60.9 (34.5–84.9)	53.1 (32.3–80.3)	0.47
PYY	231 (186–280)	240 (204–288)	0.64
PP	237 (179–342)	282 (181–473)	0.018
Glucagon	77.3 (62.4–89.1)	81.4 (65.9–98.2)	0.30
Insulin	6155 (4389–7935)	4999 (3563–8407)	0.064
C-Peptide	16,277 (13,373–17,707)	14,491 (11,037–17,817)	0.001
Amylin	79.7 (46.3–107.6)	74.1 (58.9–109.2)	0.40
Glycaemia	367 (341–425)	332 (315–358)	0.0063

## Data Availability

Data is contained within the article and supplementary material.

## Supplementary Materials

The following supporting information can be downloaded at: https://www.mdpi.com/article/10.3390/foods12040700/s1. Table S1: Anthropometrical and biochemical characteristics of the subjects recruited in the acute study (Means ± SD). Table S2: Anthropometrical and biochemical characteristics of the subjects recruited in the medium-term study (Means ± SD). Table S3: Weekly frequency of stools according to their consistency (based on the Bristol Scales) in the subjects from the control and β-glucan group during the study (Median [IQR]). Table S4: Prevalence and relative abundances of the bacterial taxa (Phylum, Family, and Genus) present in the fecal microbiota of the subjects at baseline (T0) and at the end of the treatment administration (T1) in the control and β-glucan groups. Only the taxa with a relative abundance ≥0.1% are shown. Figure S1: Total digestive symptoms by week of administration of β-glucan-enriched foods or control foods. Data distribution was normalized through square root transformation and the statistical analysis was carried out on the transformed data. Means values of these data for each group were subsequently backtransformed and are shown with their corresponding CI_95%_. Two-way ANOVA for repeated measurements: Time effect: *p* < 0.34, Treatment effect: *p* = 0.048, and Time X Treatment interaction: *p* = 0.57. Figure S2: Rarefaction curves before and after treatment with control or β-glucan supplemented foods. (Red curve: control group at T0; Blue curve: β-glucan group at T0; Orange curve: control group at T1; Green curve: β-glucan at T1).
