# Supplementary material for: Modulation of Postprandial Plasma Concentrations of Digestive Hormones and Gut Microbiota by Foods Containing Oat ß-Glucans in Healthy Volunteers
Source: Foods. 2023 Feb 6;12(4):700. doi: 10.3390/foods12040700 (PMC9955387; doi:10.3390/foods12040700)
Supplement: Supplementary file 1 [file foods-12-00700-s001.zip › Figure S1.pdf]

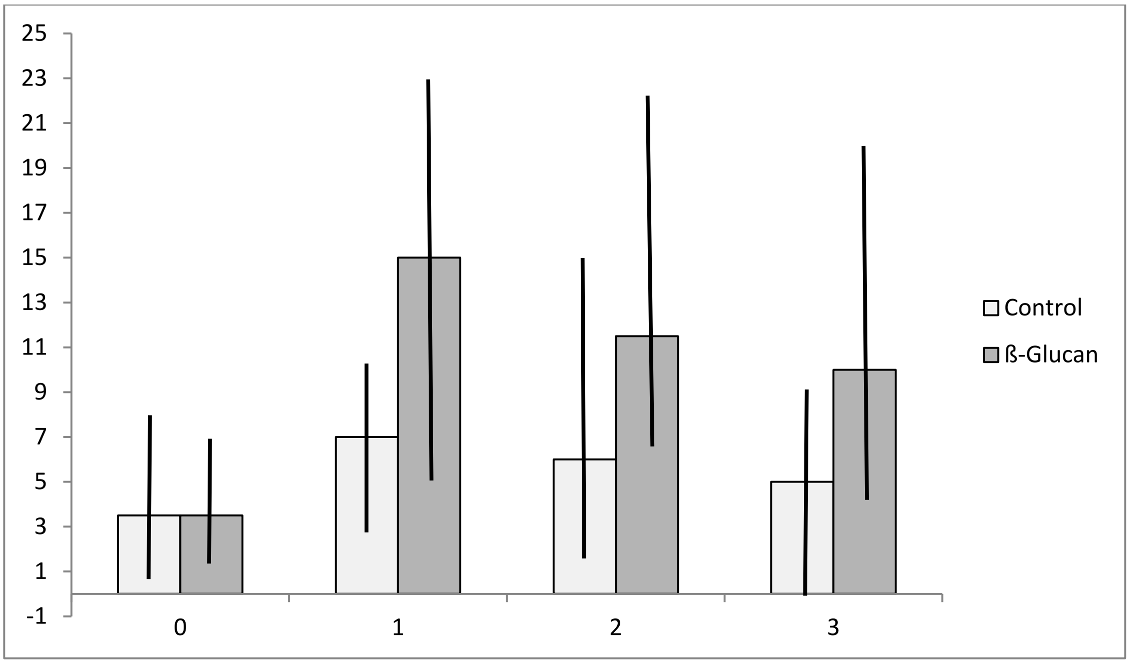

Figure S1: Total digestive symptoms by week of administration of β-glucan-enriched foods or control foods. Data distribution was normalized through square root transformation and the statistical analysis was carried out on the transformed data. Means values of these data for each group were subsequently back-transformed and are shown with their corresponding $CI_{95\%}$. Two-way ANOVA for repeated measurements: Time effect: $p<0.34$, Treatment effect: $p=0.048$, Time X Treatment interaction: $p=0.57$.
