# Supplementary material for: Modulation of Postprandial Plasma Concentrations of Digestive Hormones and Gut Microbiota by Foods Containing Oat ß-Glucans in Healthy Volunteers
Source: Foods. 2023 Feb 6;12(4):700. doi: 10.3390/foods12040700 (PMC9955387; doi:10.3390/foods12040700)
Supplement: Supplementary file 1 [file foods-12-00700-s001.zip › Figure S2.pdf]

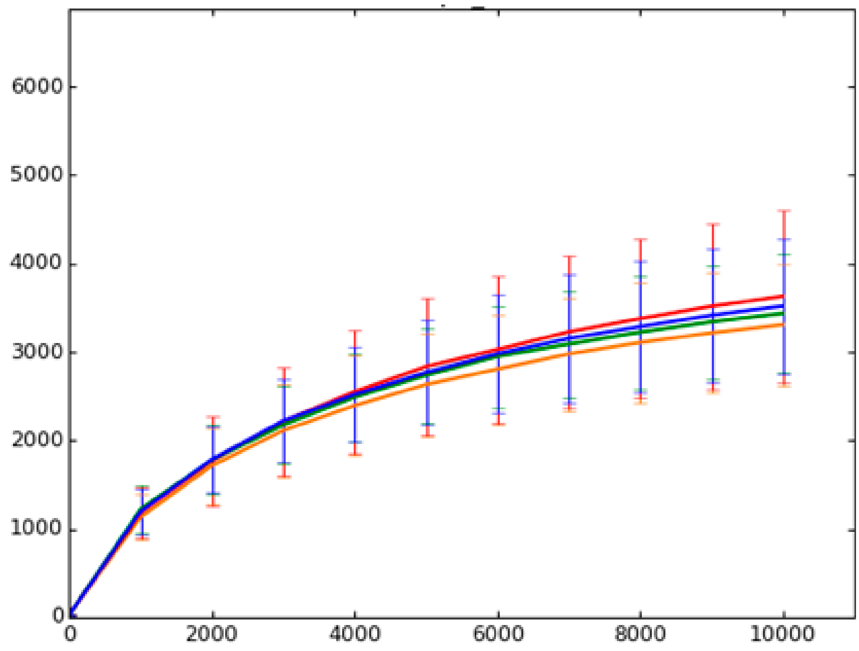

Figure S2: Rarefaction curves before and after treatment with control or β-glucan supplemented foods. (Red curve: control group at T0; Blue curve: β-glucan group at T0; Orange curve: control group at T1; Green curve: β-glucan at T1).
