# Supplementary material for: Modulation of Postprandial Plasma Concentrations of Digestive Hormones and Gut Microbiota by Foods Containing Oat ß-Glucans in Healthy Volunteers
Source: Foods. 2023 Feb 6;12(4):700. doi: 10.3390/foods12040700 (PMC9955387; doi:10.3390/foods12040700)
Supplement: Supplementary file 1 [file foods-12-00700-s001.zip › Table S1.pdf]

**Parameters**	**Values**	**Reference range**
Women (%)	85.7	
Age (y)	24,7 ± 3,2	
Body Mass Index (kg/m2)	21,6 ± 2,2	
Glycaemia (mg/dL)	93± 7	70 – 99
Ureic N_2 (mg/dL)	12,4 ± 2,4	4,7 - 23,4
Uric acid (mg/dL)	3,3 ± 0,9	2,6 - 6,0
Bilirubin (mg/dL)	0,6 ± 0,3	0,0 - 1,0
Alkaline phosphatase (U/L)	135 ± 37	65 – 300
GOT (U/L)	20,1 ± 3,1	0 – 32
LDH (U/L)	287 ± 56	230 – 460
Calcium (mg/dL)	9,9 ± 1,0	8,5 - 10,5
Phosphorus (mg/dL)	4,1 ± 0,4	2,5 - 5,6
Albumin (g/dL)	4,4 ± 0,2	3,5 - 5,2
Total protein (g/dL)	7,3 ± 0,4	6,1 - 7,9
Total cholesterol (md/dL)	194 ± 29	Hasta 200
HDL cholesterol (mg/dL)	66 ± 19	30 – 85
LDL-cholesterol (mg/dL)	109 ± 25	0 – 150
VLDL-cholesterol (mg/dL)	19 ± 5	< 40
Triacylglycerol (mg/dL)	96 ± 27	10 - 160

Table S1: Anthropometrical and biochemical characteristics of the subjects recruited in the Acute study (Means ± SD)
