# Supplementary material for: Modulation of Postprandial Plasma Concentrations of Digestive Hormones and Gut Microbiota by Foods Containing Oat ß-Glucans in Healthy Volunteers
Source: Foods. 2023 Feb 6;12(4):700. doi: 10.3390/foods12040700 (PMC9955387; doi:10.3390/foods12040700)
Supplement: Supplementary file 1 [file foods-12-00700-s001.zip › Table S2.pdf]

	**Control (n=15)**	**ß-Glucan (n=17)**	**p**
Women (%)	66.6	64.7	0.91 (X_2)
Age (y)	26.7 ± 2.8	27.1 ± 4.3	0.73
Body Mass Index (kg/m2)	20.7 ± 6.2	21.6 ± 2.3	0.54
Glycemia (mg/dL)	88.7 ± 3.8	95.7 ± 8.3	0.008
Ureic N_2 (mg/dL)	14.5 ± 4.6	15.0 ± 2.9	0.72
Uric acid (mg/dL)	3.43 ± 1.18	3.70 ± 1.24	0.55
Bilirubina total (mg/dL)	0.58 ± 0.53	0.49 ± 0.18	0,55
Alkaline phosphatase (U/L)	157 ± 45	147 ± 42	0,55
GOT (U/L)	19.5 ± 3.9	19.6 ± 3.8	0,91
LDH (U/L)	306 ± 74	326 ± 32	0,35
Calcium (mg/dL)	9.66 ± 0.44	9.83 ± 1.03	0,57
Phosforus (mg/dL)	4.39 ± 0.41	3.71 ± 0.51	0,001
Albumin (g/dL)	4.24 ± 0.15	4.32 ± 0.33	0,05
Total Protein (g/dL)	7.01 ± 0.32	7.32 ± 0.48	0,39
CS Total	182 ± 28	167 ± 24	0,14
CS HDL	63.8 ± 15.8	54.3 ± 12.8	0,09
CS LDL	96.8 ± 20.6	92.7 ± 24.9	0,64
TG	106 ± 45	97.9 ± 59.2	0,68
CS VLDL	21.2 ± 8.9	19.6 ± 11.8	0,69
CS/HDL	2.99 ± 0.80	3.14 ± 0.74	0,59

Table S2: Anthropometrical and biochemical characteristics of the subjects recruited in the Medium-term study (Means ± SD)
