# Supplementary material for: Modulation of Postprandial Plasma Concentrations of Digestive Hormones and Gut Microbiota by Foods Containing Oat ß-Glucans in Healthy Volunteers
Source: Foods. 2023 Feb 6;12(4):700. doi: 10.3390/foods12040700 (PMC9955387; doi:10.3390/foods12040700)
Supplement: Supplementary file 1 [file foods-12-00700-s001.zip › Table S3.pdf]

Stool consistency according to Bristol Scale	Time	Placebo	β-glucan	p
Hard stools (Score 1+2)	Week 1<br>Week 2<br>Week 3	1 [0 - 3]<br>0 [0 - 4]<br>0 [0 - 3]	0 [0 - 1]<br>0 [0 - 2]<br>0 [0 - 3]	NS
Normal stools (Score 3+4+5)	Week 1<br>Week 2<br>Week 3	6 [4 - 8]<br>6 [5 - 8]<br>7 [4 - 9]	7 [4 - 10]<br>7 [4 - 10]<br>7 [5 - 9]	NS
Watery stools (Score 6+7)	Week 1<br>Week 2<br>Week 3	0 [0 - 1]<br>0 [0 - 1]<br>0 [0 - 1]	0 [0 - 1]<br>0 [0 - 1]<br>0 [0 - 0]	NS

Table S3: Weekly frequency of stools according to their consistency (based on the Bristol Scales) in the subjects from the Placebo and β-glucan group during the study (Median [IQR]).
