# Supplementary material for: Modulation of Postprandial Plasma Concentrations of Digestive Hormones and Gut Microbiota by Foods Containing Oat ß-Glucans in Healthy Volunteers
Source: Foods. 2023 Feb 6;12(4):700. doi: 10.3390/foods12040700 (PMC9955387; doi:10.3390/foods12040700)
Supplement: Supplementary file 1 [file foods-12-00700-s001.zip › Table S4.pdf]

			Placebo				β-Glucan			
			Time "0"		Time "1"		Time "0"		Time "1"	
Phylum	Family	Genus	Prevalence	Mean +/- SD	Prevalence	Mean +/- SD	Prevalence	Mean +/- SD	Prevalence	Mean +/- SD
Actinobacteria			1,0	6,8 +/- 11,3	1,0	1,4 +/- 1,9	1,0	4,8 +/- 5,0	1,0	0,9 +/- 0,8
	Actinomycetaceae		0,6	0,0 +/- 0,0	0,1	0,0 +/- 0,0	0,4	0,0 +/- 0,0	0,1	0,0 +/- 0,0
		Actinomyces	0,6	0,0 +/- 0,0	0,1	0,0 +/- 0,0	0,4	0,0 +/- 0,0	0,1	0,0 +/- 0,0
	Bifidobacteriaceae		0,9	5,7 +/- 10,6	0,9	1,2 +/- 1,9	0,9	2,6 +/- 2,8	0,9	0,6 +/- 0,8
		Bifidobacterium	0,9	5,7 +/- 10,6	0,9	1,2 +/- 1,9	0,9	2,6 +/- 2,8	0,9	0,6 +/- 0,8
	Coriobacteriaceae		1,0	1,1 +/- 1,0	1,0	0,2 +/- 0,2	1,0	2,2 +/- 3,5	1,0	0,3 +/- 0,2
		Senegalimassilia	0,9	0,4 +/- 0,5	0,9	0,1 +/- 0,1	1,0	1,0 +/- 1,2	0,9	0,1 +/- 0,1
		Adlercreutzia	0,7	0,2 +/- 0,5	0,6	0,0 +/- 0,1	0,7	0,2 +/- 0,2	0,7	0,1 +/- 0,1
		Collinsella	0,7	0,3 +/- 0,6	0,5	0,0 +/- 0,0	0,7	1,0 +/- 2,5	0,5	0,0 +/- 0,1
		Eggerthella	0,5	0,1 +/- 0,3	0,3	0,0 +/- 0,0	0,4	0,0 +/- 0,0	0,3	0,0 +/- 0,0
		Slackia	0,5	0,1 +/- 0,1	0,2	0,0 +/- 0,0	0,5	0,1 +/- 0,2	0,4	0,0 +/- 0,1
Bacteroidetes			1,0	24,7 +/- 9,3	1,0	37,3 +/- 11,1	1,0	31,4 +/- 9,0	1,0	43,6 +/- 12,0
	Porphyromonadaceae		0,1	0,5 +/- 1,7	0,2	0,8 +/- 2,9	0,3	0,0 +/- 0,0	0,1	0,1 +/- 0,3
		NI	0,1	0,5 +/- 1,7	0,2	0,8 +/- 2,9	0,3	0,0 +/- 0,0	0,1	0,1 +/- 0,3
	[Barnesiellaceae]		0,9	0,5 +/- 0,6	0,9	1,2 +/- 1,3	0,7	0,4 +/- 0,6	0,8	1,0 +/- 1,3
		Barnesiella	0,9	0,5 +/- 0,6	0,9	1,2 +/- 1,3	0,7	0,4 +/- 0,6	0,8	1,0 +/- 1,3
	[Odoribacteraceae]		0,9	0,2 +/- 0,2	0,9	0,4 +/- 0,4	0,9	0,2 +/- 0,2	1,0	0,4 +/- 0,8
		Butyricimos	0,8	0,1 +/- 0,1	0,7	0,2 +/- 0,3	0,7	0,1 +/- 0,1	0,7	0,1 +/- 0,1
		Odoribacter	0,9	0,1 +/- 0,1	0,9	0,2 +/- 0,2	0,9	0,1 +/- 0,1	1,0	0,3 +/- 0,7
	[Paraprevotellaceae]		0,7	0,3 +/- 0,4	0,5	0,8 +/- 2,1	0,7	0,5 +/- 0,7	0,7	0,7 +/- 1,2
		NI	0,3	0,1 +/- 0,2	0,3	0,1 +/- 0,2	0,1	0,0 +/- 0,0	0,1	0,0 +/- 0,1
		[Prevotella]	0,3	0,1 +/- 0,3	0,1	0,5 +/- 2,1	0,3	0,3 +/- 0,7	0,3	0,4 +/- 1,2
		CF231	0,0	0,0 +/- 0,0	0,0	0,0 +/- 0,0	0,1	0,0 +/- 0,0	0,1	0,0 +/- 0,0
		Paraprevotella	0,3	0,1 +/- 0,3	0,3	0,2 +/- 0,4	0,5	0,2 +/- 0,3	0,6	0,3 +/- 0,4
	Bacteroidaceae		1,0	17,5 +/- 11,1	1,0	26,7 +/- 11,1	1,0	17,3 +/- 12,0	1,0	22,6 +/- 14,0
		**Bacteroides**	1,0	17,5 +/- 11,1	1,0	26,7 +/- 11,1	1,0	17,3 +/- 12,0	1,0	22,6 +/- 14,0
	Porphyromodaceae		1,0	0,8 +/- 0,5	1,0	1,5 +/- 0,9	1,0	0,7 +/- 0,6	1,0	1,0 +/- 0,6
		Parabacteroides	1,0	0,8 +/- 0,5	1,0	1,5 +/- 0,9	1,0	0,7 +/- 0,6	1,0	1,0 +/- 0,6
	Prevotellaceae		0,6	4,2 +/- 7,4	0,7	4,9 +/- 9,6	0,7	10,3 +/- 14,4	0,7	15,2 +/- 21,7
		Prevotella	0,6	4,2 +/- 7,4	0,7	4,9 +/- 9,6	0,7	10,3 +/- 14,4	0,7	15,2 +/- 21,7
	Rikenellaceae		0,9	0,5 +/- 1,2	1,0	0,7 +/- 1,1	0,9	0,4 +/- 0,4	1,0	1,0 +/- 1,3
		Alistipes	0,9	0,5 +/- 1,2	1,0	0,7 +/- 1,1	0,9	0,4 +/- 0,4	1,0	1,0 +/- 1,3
	S24-7		0,3	0,3 +/- 1,1	0,3	0,2 +/- 0,8	0,5	1,6 +/- 5,1	0,5	1,6 +/- 3,4
		NI	0,3	0,3 +/- 1,1	0,3	0,2 +/- 0,8	0,5	1,6 +/- 5,1	0,5	1,6 +/- 3,4
Cyanobacteria			0,3	0,1 +/- 0,2	0,6	0,4 +/- 0,8	0,4	0,1 +/- 0,3	0,5	0,2 +/- 0,6
	NI		0,3	0,1 +/- 0,2	0,5	0,3 +/- 0,8	0,4	0,1 +/- 0,3	0,4	0,2 +/- 0,6
		NI	0,3	0,1 +/- 0,2	0,5	0,3 +/- 0,8	0,4	0,1 +/- 0,3	0,4	0,2 +/- 0,6
	Chloroplast		0,0	0,0 +/- 0,0	0,3	0,0 +/- 0,0	0,0	0,0 +/- 0,0	0,1	0,0 +/- 0,0
		Streptophyta	0,0	0,0 +/- 0,0	0,3	0,0 +/- 0,0	0,0	0,0 +/- 0,0	0,1	0,0 +/- 0,0

Table S4: Prevalence and relative abundances of the bacterial taxa (Phylum, Family and Genus) present in the fecal microbiota of the subjects at baseline (T0) and at the end of the treatment administration (T1) in the Placebo and β-glucan groups. Only are shown the taxa with a relative abundance ≥ 0.1%.

	Placebo				β-Glucan			
	Time "0"		Time "1"		Time "0"		Time "1"	
Phylum / C / Family / Genus	Prevalence	Mean +/- SD	Prevalence	Mean +/- SD	Prevalence	Mean +/- SD	Prevalence	Mean +/- SD
Euryarchaeota	0,4	0,6 +/- 1,0	0,4	0,0 +/- 0,1	0,3	0,1 +/- 0,3	0,1	0,0 +/- 0,0
Methanobacteriaceae	0,4	0,6 +/- 1,0	0,4	0,0 +/- 0,1	0,3	0,1 +/- 0,3	0,1	0,0 +/- 0,0
Methanobrevibacter	0,4	0,6 +/- 1,0	0,4	0,0 +/- 0,1	0,3	0,1 +/- 0,3	0,1	0,0 +/- 0,0
Firmicutes	1,0	65,3 +/- 11,6	1,0	56,6 +/- 11,7	1,0	59,2 +/- 11,5	1,0	50,2 +/- 15,2
Lactobacillales	0,2	0,0 +/- 0,0	0,1	0,0 +/- 0,0	0,3	0,0 +/- 0,0	0,0	0,0 +/- 0,0
Lactobacillus	0,2	0,0 +/- 0,0	0,1	0,0 +/- 0,0	0,3	0,0 +/- 0,0	0,0	0,0 +/- 0,0
Enterococcaceae	0,2	0,0 +/- 0,1	0,1	0,0 +/- 0,0	0,2	0,0 +/- 0,0	0,2	0,0 +/- 0,0
Enterococcus	0,1	0,0 +/- 0,1	0,1	0,0 +/- 0,0	0,1	0,0 +/- 0,0	0,2	0,0 +/- 0,0
Lactobacillaceae	0,2	0,0 +/- 0,1	0,1	0,0 +/- 0,0	0,3	0,0 +/- 0,0	0,1	0,0 +/- 0,0
Lactobacillus	0,2	0,0 +/- 0,1	0,1	0,0 +/- 0,0	0,3	0,0 +/- 0,0	0,1	0,0 +/- 0,0
Leuconostocaceae	0,1	0,0 +/- 0,0	0,0	0,0 +/- 0,0	0,0	0,0 +/- 0,0	0,1	0,0 +/- 0,2
Weissella	0,1	0,0 +/- 0,0	0,0	0,0 +/- 0,0	0,0	0,0 +/- 0,0	0,1	0,0 +/- 0,2
Streptococcaceae	1,0	0,4 +/- 0,6	0,8	0,1 +/- 0,1	0,8	0,4 +/- 0,8	0,8	0,1 +/- 0,2
Lactococcus	0,2	0,1 +/- 0,3	0,2	0,0 +/- 0,0	0,3	0,0 +/- 0,1	0,1	0,0 +/- 0,0
Streptococcus	1,0	0,3 +/- 0,4	0,7	0,1 +/- 0,1	0,8	0,3 +/- 0,7	0,8	0,1 +/- 0,2
Turicibacteraceae	0,4	0,0 +/- 0,1	0,3	0,0 +/- 0,1	0,2	0,0 +/- 0,0	0,2	0,0 +/- 0,1
Turicibacter	0,4	0,0 +/- 0,1	0,3	0,0 +/- 0,1	0,2	0,0 +/- 0,0	0,2	0,0 +/- 0,1
Unclassified Clostridiales	1,0	4,5 +/- 3,3	1,0	4,2 +/- 3,3	1,0	3,2 +/- 1,8	1,0	3,9 +/- 2,7
**Unclassified Clostridiales**	1,0	4,5 +/- 3,3	1,0	4,2 +/- 3,3	1,0	3,2 +/- 1,8	1,0	3,9 +/- 2,7
Clostridiales_Incertae Sedis XIII	0,9	0,2 +/- 0,2	0,7	0,1 +/- 0,1	0,9	0,1 +/- 0,1	0,9	0,1 +/- 0,1
Anaerovorax	0,9	0,2 +/- 0,2	0,7	0,1 +/- 0,1	0,9	0,1 +/- 0,1	0,9	0,1 +/- 0,1
Christensenellaceae	0,8	0,3 +/- 0,6	0,7	0,2 +/- 0,6	0,8	0,2 +/- 0,2	1,0	0,3 +/- 0,5
NI	0,8	0,3 +/- 0,6	0,7	0,2 +/- 0,6	0,8	0,2 +/- 0,2	1,0	0,3 +/- 0,5
Christensenella	0,2	0,0 +/- 0,0	0,1	0,0 +/- 0,0	0,1	0,0 +/- 0,0	0,2	0,0 +/- 0,0
Clostridiaceae	1,0	1,4 +/- 1,0	1,0	0,8 +/- 1,0	1,0	1,2 +/- 0,9	1,0	0,7 +/- 0,5
NI	1,0	0,3 +/- 0,4	1,0	0,3 +/- 0,3	1,0	0,4 +/- 0,3	0,9	0,3 +/- 0,2
Clostridium	1,0	0,4 +/- 0,6	1,0	0,2 +/- 0,2	1,0	0,2 +/- 0,2	0,9	0,2 +/- 0,2
SMB53	0,5	0,0 +/- 0,0	0,3	0,0 +/- 0,0	0,5	0,0 +/- 0,0	0,3	0,0 +/- 0,0
Dehalobacteriaceae	0,1	0,0 +/- 0,0	0,1	0,0 +/- 0,0	0,1	0,0 +/- 0,0	0,4	0,0 +/- 0,0
Dehalobacterium	0,1	0,0 +/- 0,0	0,1	0,0 +/- 0,0	0,1	0,0 +/- 0,0	0,4	0,0 +/- 0,0
Lachnospiraceae	1,0	24,3 +/- 6,2	1,0	19,0 +/- 8,0	1,0	24,0 +/- 7,3	1,0	18,7 +/- 10,0
**Lachnospiracea_incertae_**	1,0	10,1 +/- 3,9	1,0	9,2 +/- 3,7	1,0	10,8 +/- 4,2	1,0	8,5 +/- 4,8
Aerostipes	0,9	0,2 +/- 0,2	0,9	0,1 +/- 0,1	0,9	0,4 +/- 0,8	0,9	0,2 +/- 0,2
**Blautia**	1,0	5,3 +/- 2,4	1,0	2,7 +/- 3,0	1,0	4,0 +/- 2,7	1,0	2,4 +/- 2,1
Coprococcus	1,0	3,1 +/- 1,5	1,0	3,0 +/- 2,3	1,0	3,1 +/- 0,9	1,0	3,4 +/- 3,5
**Dorea**	1,0	0,9 +/- 0,4	1,0	0,7 +/- 0,5	1,0	1,0 +/- 0,7	1,0	0,6 +/- 0,5
Lachnobacterium	0,0	0,0 +/- 0,0	0,1	0,0 +/- 0,0	0,1	0,0 +/- 0,0	0,1	0,0 +/- 0,0
**Lachnospira**	1,0	1,2 +/- 1,4	1,0	1,0 +/- 0,6	1,0	1,2 +/- 0,8	1,0	0,7 +/- 0,5
Roseburia	1,0	0,5 +/- 0,5	1,0	0,7 +/- 0,7	1,0	0,6 +/- 0,7	1,0	0,5 +/- 0,5
Peptococcaceae	0,3	0,0 +/- 0,1	0,1	0,1 +/- 0,2	0,3	0,0 +/- 0,0	0,3	0,0 +/- 0,0
NI	0,1	0,0 +/- 0,0	0,1	0,0 +/- 0,0	0,1	0,0 +/- 0,0	0,1	0,0 +/- 0,0
Peptococcus	0,2	0,0 +/- 0,1	0,1	0,1 +/- 0,2	0,1	0,0 +/- 0,0	0,1	0,0 +/- 0,0
Peptostreptococcaceae	0,7	0,0 +/- 0,0	0,5	0,0 +/- 0,0	0,7	0,0 +/- 0,0	0,5	0,0 +/- 0,0
NI	0,7	0,0 +/- 0,0	0,5	0,0 +/- 0,0	0,7	0,0 +/- 0,0	0,5	0,0 +/- 0,0
Ruminococcaceae	1,0	30,0 +/- 7,2	1,0	27,5 +/- 7,3	1,0	27,1 +/- 7,0	1,0	23,8 +/- 8,1
**Faecalibacterium**	1,0	13,3 +/- 4,0	1,0	12,1 +/- 4,5	1,0	11,8 +/- 4,4	1,0	10,0 +/- 4,3
Aerotruncus	0,4	0,0 +/- 0,0	0,3	0,0 +/- 0,0	0,5	0,0 +/- 0,0	0,5	0,0 +/- 0,0
**Oscillospira**	1,0	0,8 +/- 0,6	1,0	0,9 +/- 0,4	1,0	0,8 +/- 0,6	1,0	1,0 +/- 0,6
**Ruminococcus**	1,0	1,6 +/- 1,3	1,0	1,4 +/- 1,2	1,0	1,8 +/- 1,1	1,0	1,5 +/- 0,8
Veillonellaceae	1,0	1,7 +/- 1,7	1,0	1,9 +/- 2,4	1,0	1,6 +/- 1,8	1,0	1,4 +/- 0,7
Acidaminococcus	0,1	0,0 +/- 0,0	0,1	0,1 +/- 0,3	0,1	0,1 +/- 0,3	0,1	0,0 +/- 0,1
Dialister	0,7	0,9 +/- 1,0	0,7	0,9 +/- 0,8	0,7	0,8 +/- 1,0	0,8	0,9 +/- 0,8
Megamos	0,3	0,2 +/- 0,9	0,1	0,6 +/- 2,3	0,3	0,4 +/- 1,7	0,2	0,2 +/- 0,6
Megasphaera	0,1	0,4 +/- 1,5	0,1	0,0 +/- 0,1	0,1	0,0 +/- 0,0	0,1	0,0 +/- 0,0
Mitsuokella	0,0	0,0 +/- 0,0	0,0	0,0 +/- 0,0	0,1	0,0 +/- 0,1	0,1	0,0 +/- 0,0
Phascolarctobacterium	0,5	0,1 +/- 0,1	0,6	0,2 +/- 0,3	0,6	0,2 +/- 0,5	0,4	0,3 +/- 0,4
Succiniclasticum	0,1	0,0 +/- 0,2	0,0	0,0 +/- 0,0	0,1	0,0 +/- 0,0	0,0	0,0 +/- 0,0
Veillonella	0,5	0,0 +/- 0,1	0,5	0,1 +/- 0,3	0,3	0,0 +/- 0,0	0,5	0,0 +/- 0,0
Erysipelotrichaceae	1,0	1,6 +/- 3,6	1,0	1,9 +/- 3,6	1,0	0,8 +/- 0,9	0,9	0,6 +/- 0,6
Asteroleplasma	0,9	1,2 +/- 3,5	0,9	1,8 +/- 3,6	0,8	0,4 +/- 0,5	0,8	0,4 +/- 0,4
[Eubacterium]	0,5	0,3 +/- 0,6	0,5	0,1 +/- 0,1	0,6	0,2 +/- 0,5	0,5	0,2 +/- 0,4
Bulleidia	0,1	0,0 +/- 0,1	0,1	0,0 +/- 0,0	0,1	0,0 +/- 0,0	0,1	0,0 +/- 0,0
Catenibacterium	0,1	0,1 +/- 0,2	0,1	0,0 +/- 0,2	0,1	0,2 +/- 0,8	0,3	0,1 +/- 0,2
cc_115	0,1	0,0 +/- 0,0	0,0	0,0 +/- 0,0	0,1	0,0 +/- 0,0	0,1	0,0 +/- 0,0
Holdemania	0,3	0,0 +/- 0,0	0,5	0,0 +/- 0,0	0,3	0,0 +/- 0,0	0,3	0,0 +/- 0,0
Lentisphaerae	0,3	0,0 +/- 0,0	0,3	0,0 +/- 0,0	0,3	0,5 +/- 1,8	0,2	0,5 +/- 2,1
Victivallaceae	0,3	0,0 +/- 0,0	0,3	0,0 +/- 0,0	0,3	0,5 +/- 1,8	0,2	0,5 +/- 2,1
Victivallis	0,3	0,0 +/- 0,0	0,3	0,0 +/- 0,0	0,3	0,5 +/- 1,8	0,2	0,5 +/- 2,1

Table S4 (…/…)

	Placebo				β-Glucan			
	Time "0"		Time "1"		Time "0"		Time "1"	
Phylum / C / Family / Genus	Prevalence	Mean +/- SD	Prevalence	Mean +/- SD	Prevalence	Mean +/- SD	Prevalence	Mean +/- SD
Proteobacteria	1,0	0,6 +/- 0,5	1,0	2,4 +/- 3,7	1,0	3,4 +/- 9,3	1,0	3,4 +/- 8,2
Alcaligeceae	0,7	0,3 +/- 0,3	0,9	0,7 +/- 1,0	0,9	0,4 +/- 0,4	1,0	0,5 +/- 0,5
Sutterella	0,7	0,3 +/- 0,3	0,9	0,7 +/- 1,0	0,9	0,4 +/- 0,4	1,0	0,5 +/- 0,5
Oxalobacteraceae	0,1	0,0 +/- 0,0	0,3	0,0 +/- 0,0	0,0	0,0 +/- 0,0	0,0	0,0 +/- 0,0
Oxalobacter	0,1	0,0 +/- 0,0	0,3	0,0 +/- 0,0	0,0	0,0 +/- 0,0	0,0	0,0 +/- 0,0
Desulfovibrioceae	0,8	0,2 +/- 0,3	0,7	0,2 +/- 0,4	0,8	0,2 +/- 0,3	0,8	0,2 +/- 0,3
NI	0,3	0,0 +/- 0,0	0,2	0,0 +/- 0,0	0,2	0,0 +/- 0,0	0,2	0,0 +/- 0,0
Bilophila	0,7	0,1 +/- 0,1	0,7	0,0 +/- 0,1	0,7	0,1 +/- 0,1	0,6	0,1 +/- 0,1
Desulfovibrio	0,3	0,1 +/- 0,2	0,3	0,2 +/- 0,4	0,5	0,1 +/- 0,3	0,4	0,1 +/- 0,3
Succinivibrioceae	0,0	0,0 +/- 0,0	0,1	0,1 +/- 0,2	0,3	2,4 +/- 9,4	0,1	2,1 +/- 8,0
Succinivibrio	0,0	0,0 +/- 0,0	0,1	0,1 +/- 0,2	0,3	2,4 +/- 9,4	0,1	2,1 +/- 8,0
Enterobacteriaceae	0,7	0,1 +/- 0,1	0,8	0,1 +/- 0,2	0,8	0,2 +/- 0,4	0,8	0,4 +/- 0,9
genus Escherichia/Shigella	0,7	0,1 +/- 0,1	0,8	0,1 +/- 0,2	0,8	0,2 +/- 0,4	0,8	0,4 +/- 0,9
Klebsiella	0,0	0,0 +/- 0,0	0,1	0,0 +/- 0,0	0,1	0,0 +/- 0,0	0,1	0,0 +/- 0,0
Pasteurellaceae	0,4	0,0 +/- 0,1	0,6	0,7 +/- 2,6	0,3	0,0 +/- 0,0	0,5	0,0 +/- 0,1
Haemophilus	0,4	0,0 +/- 0,1	0,6	0,7 +/- 2,6	0,3	0,0 +/- 0,0	0,5	0,0 +/- 0,1
Synergistetes	0,1	0,0 +/- 0,0	0,2	0,0 +/- 0,0	0,1	0,0 +/- 0,0	0,1	0,0 +/- 0,0
Synergistaceae	0,1	0,0 +/- 0,0	0,2	0,0 +/- 0,0	0,1	0,0 +/- 0,0	0,1	0,0 +/- 0,0
Tenericutes	0,4	0,1 +/- 0,3	0,4	0,1 +/- 0,3	0,2	0,0 +/- 0,0	0,5	0,0 +/- 0,1
assimiled to molicutes order	0,3	0,1 +/- 0,1	0,3	0,0 +/- 0,1	0,1	0,0 +/- 0,0	0,3	0,0 +/- 0,1
assimiled to RF3 order	0,3	0,1 +/- 0,2	0,3	0,1 +/- 0,2	0,1	0,0 +/- 0,0	0,2	0,0 +/- 0,0
Verrucomicrobia	0,7	1,7 +/- 3,4	0,7	1,5 +/- 1,8	0,7	0,3 +/- 0,4	0,7	1,0 +/- 1,8
[Cerasicoccaceae]	0,2	0,0 +/- 0,0	0,1	0,0 +/- 0,0	0,1	0,0 +/- 0,0	0,0	0,0 +/- 0,0
NI	0,2	0,0 +/- 0,0	0,1	0,0 +/- 0,0	0,1	0,0 +/- 0,0	0,0	0,0 +/- 0,0
Verrucomicrobiaceae	0,7	1,7 +/- 3,4	0,7	1,5 +/- 1,8	0,7	0,3 +/- 0,4	0,7	1,0 +/- 1,8
Akkermansia	0,7	1,7 +/- 3,4	0,7	1,5 +/- 1,8	0,7	0,3 +/- 0,4	0,7	1,0 +/- 1,8

Table S4 (…/…)
